# A validated web-application (GFDC) for automatic classification of glaucomatous visual field defects using Hodapp-Parrish-Anderson criteria

**DOI:** 10.1038/s41746-024-01122-8

**Published:** 2024-05-18

**Authors:** Arun James Thirunavukarasu, Nikhil Jain, Rohan Sanghera, Federico Lattuada, Shathar Mahmood, Anna Economou, Helmut C. Y. Yu, Rupert Bourne

**Affiliations:** 1 University of Cambridge School of Clinical Medicine, University of Cambridge, Cambridge, UK; 2 Oxford University Clinical Academic Graduate School, University of Oxford, Oxford, UK; 3 Cambridge Eye Research Centre, Cambridge University Hospitals NHS Foundation Trust, Cambridge, UK; 4 Oxford University Hospitals NHS Foundation Trust, Oxford, UK; 5 Bedfordshire Hospitals NHS Foundation Trust, Luton, UK; 6 Department of Engineering, University of Cambridge, Cambridge, UK; 7 University of New South Wales, Sydney, Australia; 8 Vision & Eye Research Institute, School of Medicine, Anglia Ruskin University, Cambridge, UK

**Keywords:** Diagnosis, Prognosis, Eye manifestations, Data processing

## Abstract

Subjectivity and ambiguity of visual field classification limits the accuracy and reliability of glaucoma diagnosis, prognostication, and management decisions. Standardised rules for classifying glaucomatous visual field defects exist, but these are labour-intensive and therefore impractical for day-to-day clinical work. Here a web-application, Glaucoma Field Defect Classifier (GFDC), for automatic application of Hodapp-Parrish-Anderson, is presented and validated in a cross-sectional study. GFDC exhibits perfect accuracy in classifying mild, moderate, and severe glaucomatous field defects. GFDC may thereby improve the accuracy and fairness of clinical decision-making in glaucoma. The application and its source code are freely hosted online for clinicians and researchers to use with glaucoma patients.

## Introduction

Glaucoma is a heterogenous group of diseases characterised by cupping of the optic nerve head and visual-field damage^1^. It is the most frequent cause of irreversible blindness worldwide^2^. Interpretation of visual fields is an essential part of diagnosis, severity grading, and prognostication in glaucoma. However, clinical assessment of visual fields is unreliable due to subjectivity and ambiguity in guidance documentation^3^. This leads to differential treatment of patients based on arbitrary factors which can lead to adverse outcomes. For instance, decisions to certify patients as visually impaired depend on the severity of patients’ visual field defects which are explicitly defined as a clinical decision (rather than being based on explicit objective criteria) in United Kingdom guidelines^4^. Patients eligible for social support due to sight impairment are frequently unregistered as a consequence of such subjectivity^5–7^. Glaucoma patients eligible on the basis of visual field defects are significantly more likely to miss out on registration than patients eligible on the basis of visual acuity, because of significant disagreement between ophthalmologists evaluating visual fields using idiosyncratic and subjective clinical criteria^6,8–10^. Moreover, risk stratification of glaucoma patients is a priority when timely glaucoma care is challenged by increasing demand for services, as was highlighted during the COVID pandemic^11^. Staging glaucomatous field defects is an important component of such risk stratification^12^.

Standardised rules for classifying glaucomatous field defects were proposed by Hodapp, Parish, and Anderson in 1993, who defined ‘early’, ‘moderate’, and ‘severe’ defects based on the mean deviation, global plot, and pattern deviation on Humphrey visual field test printouts^13^. The Hodapp-Parish-Anderson (HPA) criteria have been used widely in research studies for their clarity and reproducibility, and HPA decisions align closer with glaucoma subspecialists than general ophthalmologists without specific expertise^9,14^. Moreover, the HPA criteria are less likely to underestimate the severity of glaucomatous damage than simpler global parameters such as mean deviation or the visual field index, perhaps because they incorporate mean deviation in addition to other parameters^15,16^. However, use in regular clinical practice is limited due to the labour intensive requirement to evaluate multiple parameters presented on perimetry plots for every assessment. Accelerating incorporation of HPA criteria into clinical workflows could improve the accuracy, reliability, and fairness of visual field assessment for glaucoma patients. Here, GFDC (Glaucoma Field Defect Classifier), a web-application which automates grading based on HPA criteria without requiring patient-identifiable data to be inputted, is presented and validated.

### Accuracy and practicality

**Fig. 1:**
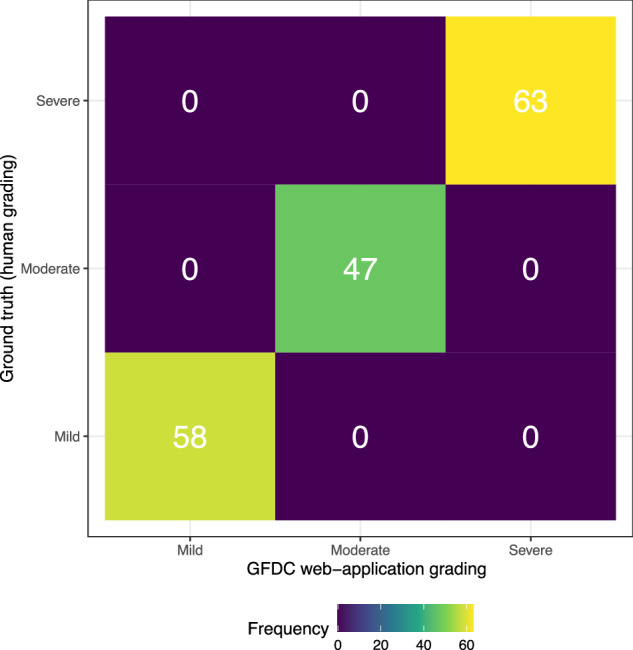
The Glaucoma Field Defect Classifier exhibits perfect accuracy. A contingency table depicting perfect agreement between glaucomatous field defect classification by human researchers (y-axis) and the Glaucoma Field Defect Classifier (GFDC; x-axis) when using Hodapp-Parrish-Anderson criteria to grade perimetry results. In every case, GFDC reached the same conclusion as the ground truth defined by human researchers, indicating perfect accuracy.

For every perimetry result, GFDC web-application output matched the ground truth defined by human researchers applying HPA criteria (Fig. 1). As no fields with glaucomatous field defects were classified as having no defect, and no severe defects were classified as exhibiting a mild or moderate defect, the sensitivity of detecting any glaucomatous field defect and a severe glaucomatous field defect was 100%. Specificity for detecting severe glaucomatous field defects was also 100%. As no fields exhibited no field defect, the specificity of detecting any glaucomatous field defect was undefined. Accuracy overall and F1 score for detecting severe glaucomatous field defect were 100%. Agreement between human graders (ground truth) and GFDC was perfect (κ = 1.00). Blinded human researchers appraising the same plots exhibited disagreement in 5 cases (κ = 0.97).

Perimetry appraisal using GFDC was significantly faster than manual application of HPA criteria (Table 1). However, the duration of manual appraisal exhibited greater variation, with some manual assessments being faster than any GFDC-facilitated assessment. Researchers commented that they did not need to apply every HPA criterion in some cases, such as where mean deviation was sufficient to grade a field defect as severe irrespective of other parameters. Allowing researchers to make similar judgements when using GFDC—for instance, instantly interpreting mean deviation readings less than -20 dB as severe rather than inputting all parameters into GFDC—would likely decrease the average duration and increase the variability of duration of GFDC-facilitated appraisal in a similar fashion.

**Table 1:** The Glaucoma Field Defect Classifier facilitates faster visual field interpretation with less variance than manual classification

Schema	Average duration ± standard deviation (s)	*t* statistic	*p* value
GFDC	26.5 ± 3.3	−3.46	0.003
Manual	48.6 ± 28.4		

Comparative duration of perimetry plot evaluation through manual application of Hodapp-Parrish-Anderson criteria, and through use of the GFDC web-application. Although manual classification was sometimes faster than using the Glaucoma Field Defect Classifier (GFDC), GFDC was faster on average (*p* = 0.003). Manual appraisal exhibited higher variability due to researchers being able to ignore redundant steps of the classification algorithm at their discretion.

### Interpretability and explainability

**Fig. 2:**
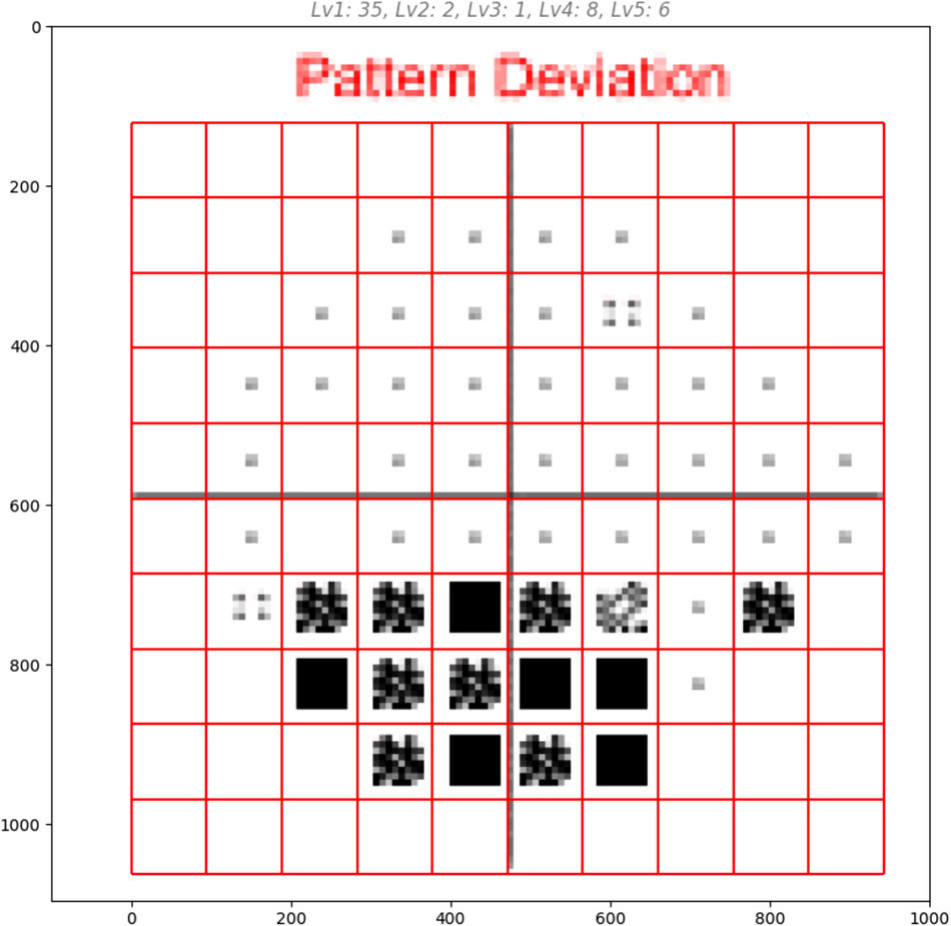
The Glaucoma Field Defect Classifier mechanism of operation is interpretable. A screenshot taken from model diagnostics and interpretability work undertaken during development of the Glaucoma Field Defect Classifier (GFDC). A computer vision algorithm recognises the bounds of the pattern deviation plot and superimposes a grid to capture test loci, before categorising pattern deviation at each locus to facilitate count computation and application of Hodapp-Parrish-Anderson criteria. The same schema may be applied to work with different plot sizes, colour schemes, legends, and perimetry parameters such as total points (e.g. 30-2 rather than 24-2 SITA), and defined significance levels. Alternative classification criteria may also be incorporated.

Simple thresholds for mean deviation and central global plot decibel values (detailed in Table 2) are explicitly coded into the algorithm. To interpret pattern deviation plots, a computer vision algorithm is designed to identify plot boundaries and result points as shown in Fig. 1. A matrix is generated based on the pattern deviation identified at each result point (Fig. 2), which is then used to apply encoded HPA criteria described in Table 1.

**Table 2:** The Hodapp-Parrish-Anderson criteria used by the Glaucoma Field Defect Classifier

Variable	General rule
Mean deviation	≥-1 dB: no defect
	-1 dB & ≥-6 dB: mild defect
	-6 dB & ≥-12 dB: moderate defect
	> 12 dB: severe defect
Central 5° points on global plot	All >15 dB: no defect
	Any points (not in both hemifields) dB: moderate defect
	Points in both hemifields dB: severe defect
	Any points =0 dB: severe defect
Pattern deviation plot (proportion of points depressed below *P* < 5%)	0%: no defect
	>0% & ≤25%: mild defect
	>25% & ≤50%: moderate defect
	>50%: severe defect
Pattern deviation plot (proportion of points depressed below *P* < 1%)	0: no defect
	>0 & <10/76: mild defect
	≤10/76 & 20/76: moderate defect
	≤20/76: severe defect

Hodapp-Parrish-Anderson (HPA) criteria distilled into a concise algorithm for human researchers to use and for transposition into computer code for automated grading. Proportion of pattern deviation points is used in the specific criteria because the absolute number of points differs between test schema (e.g. 24-2, 30-2). Where criteria provide different results, the most severe result is taken as the overall grading (in-keeping with the original HPA schema).

The web-application validated here has the potential to facilitate incorporation of HPA-based visual field assessment at scale. 100% sensitivity for detection of any glaucomatous field defect and a severe field defect suggests that no patients would be dismissed as a false negative by GFDC, maximising safety with deployment. 100% specificity for detecting severe field defects indicates that the algorithm can identify patients at high risk or with significant deficits without compromising efficiency by including other patients as false positives. Adopting standard criteria for visual field appraisal in glaucoma clinics would ameliorate one of the most severe sources of arbitrary discrepancy in diagnosis, assessment of progression, prognosis, and identification of vision impairment.^6,7,14^ Using an explicitly coded computer vision algorithm reduces the time-requirement for clinicians to leverage validated criteria, overcomes black box limitations associated with machine learning approaches, and minimises the possibility of erroneous decisions made for uninterpretable reasons^17^. Another application may be in providing a more precise and systematic approach to the grading of visual fields required by some countries’ social service benefit criteria. For example, an automated HPA interpretation of an integrated binocular visual field may offer more specificity than the visual field index (as used in some countries), in judging visual function or disability.

Two limitations should be considered alongside potential applications of the web-application. First, HPA criteria may overestimate or underestimate glaucomatous damage, and alternative criteria may be more appropriate for clinical practice^9,15,18^. Second, different perimetry machines may display results in formats incompatible with the code used above. Both limitations may be ameliorated by simple modifications to the published code, which can be easily adapted to adjust criteria for classification, incorporate other classification schemata, or work with alternative results formats. Subsequent work is underway using GFDC to explore the clinical utility of automated HPA-based appraisal of glaucoma patients. External research teams are welcome to use and adapt the code and web-application for the benefit of patients and ophthalmologists. Reducing subjectivity of perimetry analysis without compromising clinical accuracy and precision may help ensure glaucoma patients receive equitable and optimal care.

## Methods

### Web-application development

HPA criteria were distilled into a three-level algorithm corresponding to the format of the 24-2 Humphrey visual field results obtained at a tertiary centre for glaucoma (Table 2)^13,19^. The algorithm was transposed into Python code accepting three inputs: mean deviation, central four decibel (dB) readings from the global plot, and pattern deviation plot. OpenCV was used to convert pattern deviation plots into matrices with identities corresponding to levels of pattern deviation^20^. A graphical-user interface was developed in CSS, JavaScript, and HTML, and deployed online for researchers to use (https://gfdc.app). Code is publicly available on a GitHub repository (https://github.com/RohanSanghera/gfdc).

### Validation

To validate the accuracy of the web-application, its output was compared to human clinical researchers using the same Hodapp-Parish-Anderson criteria (Table 1). 168 consecutively recorded visual fields from glaucoma clinics were used for the study. Each researcher graded 30-40 visual fields, with a total of 168 eyes from 89 patients comprising the validation dataset. To mitigate human error, every visual field was evaluated by two independent researchers, with disagreement resolved by a third researcher acting as arbiter. These human decisions were accepted as gold-standard ground truth and used to define whether the web-application was correct or not for every visual field in the dataset.

To evaluate the practicality of GFDC, a single researcher conducted screening on consecutive records with conventional manual and novel application-facilitated methods in randomised fashion: evaluation method was determined by a coin flip until both methods had been used at least 20 times. A blinded, independent researcher measured the time required to generate a final grading.

This retrospective study comprised part of an ongoing service improvement project (ID4167) granted approval by the Cambridge University Hospitals NHS Foundation Trust Audit Department (PRN10167).

### Statistical analysis

Agreement between human researchers and the web-application was quantified through calculation of a Kappa statistic. Confusion matrix analysis was also employed to evaluate the overall accuracy of the web-application in identifying patients with any visual field defect, and a ‘severe’ visual field defect (which would correspond to automatic eligibility for sight impairment certification based on UK guidelines)^4^. To determine if there was any difference between the duration of appraisal with and without GFDC, a *t*-test was computed with *p* = 0.05 taken as the accepted level of statistical significance. Statistical analysis and data visualisation were conducted in R (version 4.1.2; R Foundation for Statistical Computing, Vienna, Austria).

### Reporting summary

Further information on research design is available in the Nature Research Reporting Summary linked to this article.

### Supplementary information

Reporting Summary

## Data Availability

All data required to replicate analyses are available from the authors.

## Code availability

All code required to implement GFDC is publically available on a GitHub repository at the following link: https://github.com/RohanSanghera/gfdc.
